# Varicocele in an Adult Ram: Histopathological Examination and Sperm Quality Evaluation

**DOI:** 10.3390/vetsci9020086

**Published:** 2022-02-17

**Authors:** Athina Basioura, Georgia D. Brellou, Ioannis Tsakmakidis, Ioannis Vlemmas, Constantin M. Boscos, Nektarios Giadinis

**Affiliations:** 1 Department of Agriculture, School of Agricultural Sciences, University of Western Macedonia, 53100 Florina, Greece; abasioura@uowm.gr; 2 Department of Pathology, School of Veterinary Medicine, Faculty of Health Sciences, Aristotle University of Thessaloniki, 56427 Thessaloniki, Greece; mprellou@vet.auth.gr (G.D.B.); ivlemmas@vet.auth.gr (I.V.); 3 Farm Animals Clinic, School of Veterinary Medicine, Faculty of Health Sciences, Aristotle University of Thessaloniki, 54627 Thessaloniki, Greece; pboscos@vet.auth.gr

**Keywords:** ram, spermatozoa, testis, varicocele

## Abstract

Varicocele is a common pathological condition of testis that is related to male fertility problems. A 3-year age Chios ram had an abnormally enlarged scrotal area, was excluded from reproductive duties, and was euthanized with the owners’ permission. The main pathological finding was the presence of bilateral multinodular spermatic cord enlargement with laminated vascular thrombi. Histopathological examination revealed commonly mineralized thrombi within the lumen of veins of the pampiniform plexus, inflammation and testicular degeneration. The epididymides were transported to the laboratory and each cauda region was sliced and washed (8 mL water for injection/epididymis), and the epididymal sperm samples were collected. Sperm motility variables (CASA), viability (eosin-nigrosine), morphology (SpermBlue^®^), and DNA integrity (Acridine Orange Test, AOT) were assessed. The total and progressive motility were low in semen samples of both sides (30.00% and 1.00% vs. 42.60% and 2.50% for left and right epididymis, respectively). Low viability values were observed for both sides (26.00% vs. 23.00% for left and right epididymis, respectively), while sperm morphological abnormalities were within normal limits. No sperm with DNA damage were detected. The results of this case report indicate that varicocele is associated with testis dysfunction and degradation of ram semen quality, mainly affecting motility and kinematics.

## 1. Introduction

Varicocele is a testicular pathological condition with a characteristic abnormal dilation, enlargement and tortuosity of veins in the pampiniform plexus, which is associated with male sub- or infertility [1,2]. It is a common disorder of the male reproductive system, with many recent studies shedding light on diagnosis based on the use of potential biomarkers [3] or therapeutic protocols, including surgical procedures [4]. Three possible causes of male varicocele have been reported: (i) the anatomical differences between the right and left sperm vein, which lead to a high hydrostatic pressure and, subsequently, to vein enlargement; (ii) the abnormal or lack of valves, which could cause a reflux of blood; (iii) “the nutcracker phenomenon”, in which the renal vein is compressed between the aorta and the upper mesenteric artery [5]. In farm animals, however, this has been less investigated or less diagnosed over the years [6,7,8]. Regarding varicocele in rams, there are few announcements, and most were published almost 40 years ago [8,9]. In rams, varicocele could be bilateral and is most common in old animals [8], in contrast to humans, in which it is usually observed in the left side, with the highest prevalence in young men [1]. Possible causes of varicocele in rams include trauma in the testis, especially for rams that are housed in groups [10], or inheritance reasons [11]. Even the pathophysiological mechanisms of infertility related to varicocele have not been completely elucidated; it is possible that the regression of blood leads to a high temperature in the testis, increased intratesticular pressure, hypoxia, and oxidative stress, which subsequently negatively affect spermatogenesis and testosterone production by Leydig cells or could cause testicular atrophy [12]. Depending on the varicocele grade, the semen parameters could be impaired, and the fertilizing ability of spermatozoa could be negatively influenced [12]. Although it is well-known that varicocele is associated with sperm damage, this issue has not been studied in rams with modern laboratory tests to elucidate how varicocele affects the qualitative semen variables.

## 2. Case Presentation

**Figure 1:**
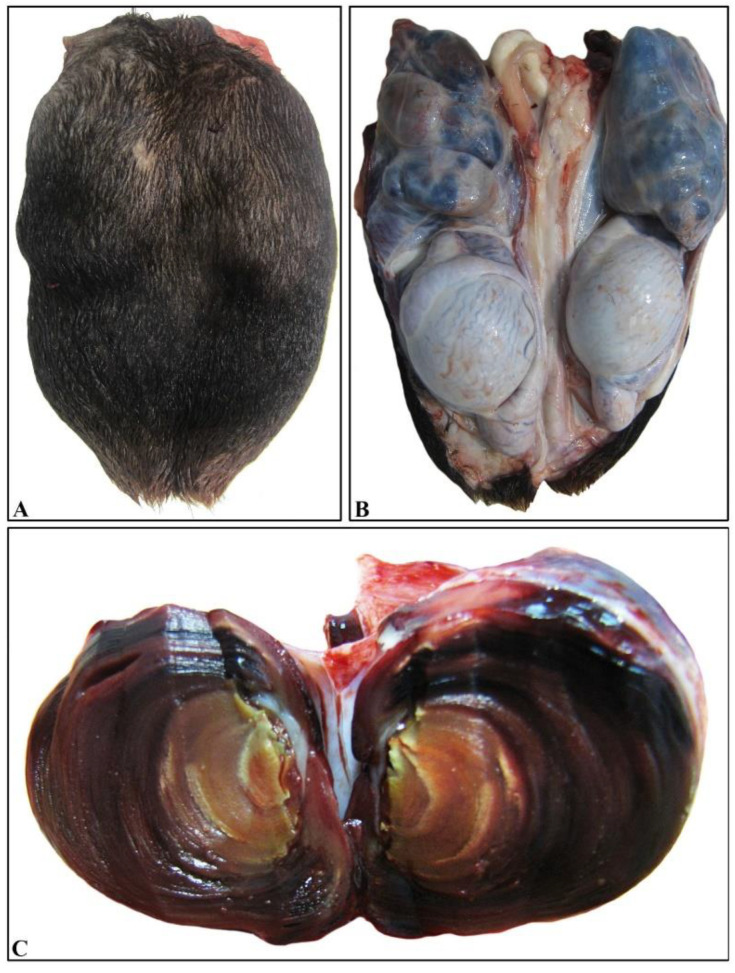
Bilateral thrombosed varicocele. (**A**) Significant enlargement of the scrotum that covers the testes. Note the uneven, nodular outline. (**B**) Scrotal incision and removal reveal relatively normal morphology both of testes and epididymides and marked enlargement of both pampiniform plexuses. The plexuses appear turgid and are characterized by tortuous shape and mostly blue color. (**C**) The cut surface of a markedly dilated vein shows the development of a large intraluminal thrombus consisting of layered concentric rings.

A 3-year-old Chios ram was admitted to the Farm Animals Clinic, School of Veterinary Medicine, Faculty of Health Sciences, Aristotle University of Thessaloniki, demonstrating an engorged scrotal area. The animal was excluded from reproductive farm duties and was slaughtered in the abattoir with the owner’s consent. The dominant pathological finding was the presence of bilateral multinodular spermatic cord enlargement attributed to vascular thrombosis. Both pampiniform venus plexuses were firm on palpation, with the left one being slightly larger than the right one. Torsion of the left testicle was also seen, which was most likely recent, since only a mild hyperemia was evident (Figure 1A,B). Each thrombus completely occluded the lumen of the vein and was partly attached to its wall. On the cut surface, thrombi presented a layered aspect (Figure 1C).

**Figure 2:**
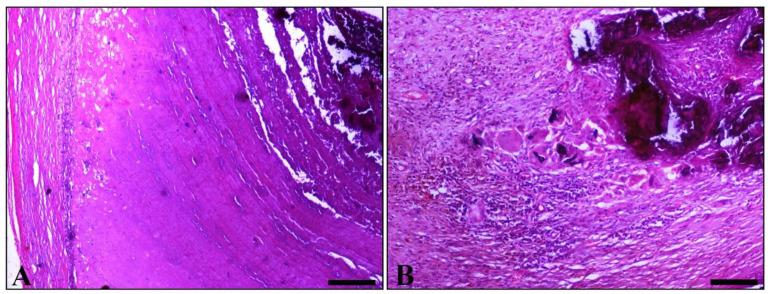
Histopathological figures of vascular thrombosis. (**A**) Part of the obstructed vein. A degenerated vessel (left) and thrombotic material, which strongly adhered to the vessels wall. In thrombus, a laminated pattern consistent with that macroscopically detected in Figure 1C predominates. The marked basophilic appearance, related to the concentration of minerals, is obvious. (**B**) Part of a degenerated pampiniform vein wall shows amorphous, intensely basophilic, mineralized, material (upper right). Adjacent to the above lesion, granulomatous inflammation is observed, mainly comprised of epithelioid macrophages and giant cells, some of which contain a phagocytosed basophilic material similar to that observed within the vessel wall. (H&E, bar, A = 250 μm, bar, B = 100 μm).

**Figure 3:**
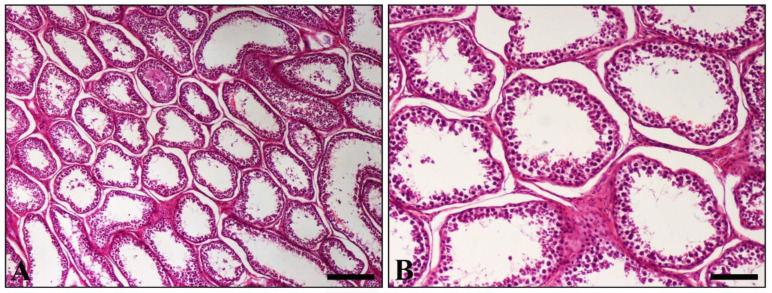
Histopathological section of testis. (**A**) Diffuse depletion and distention of seminiferous tubules. Only a few spermatogenic cells line the majority of tubules; (**B**) Higher magnification of (**A**) showing tubules depleted of all germ cells. (H&E, A = 250 μm, bar, B = 100 μm).

Histopathological examination of various sections from pampiniform plexuses revealed the presence of commonly mineralized thrombi, which occluded the lumen of veins. Vascular wall mineralization, usually accompanied by granulomatous inflammation, located mostly in the region where the thrombus is firmly attached to the wall, was also evident (Figure 2A,B). An additional finding observed in both testes was degeneration, characterized by the depletion of seminiferous tubules and absence of spermatids (Figure 3).

An hour after the animal’s death, the epididymides were transported to the Unit of Biotechnology of Reproduction (Farm Animals Clinic, School of Veterinary Medicine, Faculty of Health Sciences, Aristotle University of Thessaloniki). Before further analysis of sperm parameters, each epididymis was weighted. The left and the right epididymis weight was 27.72 g and 28.86 g, respectively. Then, each cauda region was sliced, washed with water for injection (37 °C; 8 mL/epididymis) and the epididymal sperm samples were collected in a centrifuge tube. Two semen samples were created, corresponding to the right and left epididymis. Each semen sample was assessed for sperm motility, viability, morphology and DNA integrity.

A computer-assisted sperm analysis (CASA) system (Sperm Class Analyser^®^, Microptic S.L., Automatic Diagnostic Systems, Barcelona, Spain), a microscope (AXIO Scope A1, Zeiss, Germany) equipped with a heating stage (37 °C) and a camera (Basler scA780 54fc, Ahrensburg, Germany) were used for epididymal sperm motility and kinetics assessment. For the analysis, the Sperm Class Analyzer^®^ software (SCA^®^ v.6.3.; Microptic S.L., Automatic Diagnostic Systems, Barcelona, Spain) was used, applying the following configurations: analysis of 4–6 fields (×100) for each semen sample, >500 sperm, 25 frames/s, progressive movement of >80% of the parameter STR, depth of field 10. The debris identified as spermatozoa were manually deleted. A volume of 10 μL of each semen sample was placed on the Makler chamber (10 μm deep; Makler^®^ counting chamber, Sefi Medical Instruments, Haifa, Israel), which was preheated at 37 °C, and the CASA sperm motility/kinetics were examined as follows: total/progressive motility (%); rapid, medium, slow movement spermatozoa (10 < slow < 45 < medium < 75 < rapid μm/s; %); curvilinear velocity (VCL; μm/s); straight line velocity (VSL; μm/s); average path velocity (VAP; μm/s); amplitude of lateral head displacement (ALH; μm); beat/cross-frequency (BCF; Hz); linearity (LIN; VSL/VCL × 100); straightness (STR; VSL/VAP × 100); wobble (WOB; VAP/VCL × 100).

**Figure 4:**
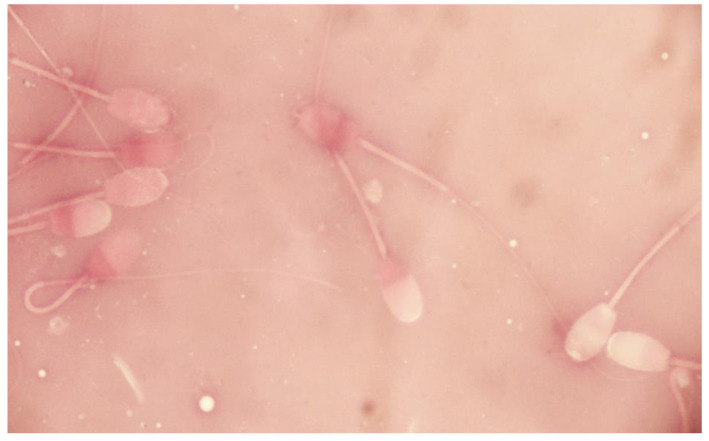
Sperm viability of epididymal ram spermatozoa (eosin-nigrosine stain).

Sperm viability was assessed using the double-staining protocol of eosin-nigrosine in one step. A total number of 200 spermatozoa per sample was assessed using a Zeiss optical microscope (Oberkochen, Germany) at magnification ×1000. Spermatozoa with intact or damaged plasma membrane were expressed as percentage of live or dead spermatozoa, respectively (Figure 4).

The SpermBlue staining method (SpermBlue^®^ 08029, Microptic S.L., Barcelona, Spain) was applied according to the manufacturer’s instructions for sperm morphology assessment. In total, 200 spermatozoa per sample were microscopically counted (×400), and the results were expressed as percentage of spermatozoa with normal morphology or with morphological abnormalities (head (including integrity of acrosome membrane), midpiece, tail, cytoplasmic droplets).

**Figure 5:**
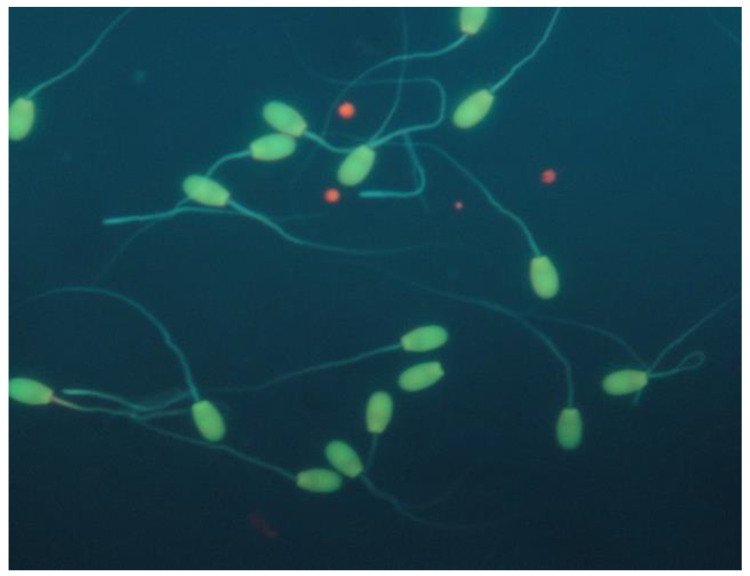
Sperm DNA integrity of epididymal ram spermatozoa (acridine orange stain).

Sperm DNA integrity was estimated by applying the Acridine Orange Test (AOT) [13]. A total of 200 spermatozoa were counted using a fluorescence microscope (×1000; Zeis, Oberkochen, Germany). The results were expressed as percentage (%) of spermatozoa with intact (sperm head fluorescent green) or fragmented (sperm head fluorescent red/orange/yellow) chromatin structure (Figure 5).

All the evaluated sperm CASA motility parameters are presented in Table 1. According to the above-mentioned analyses, the total and progressive motility were low in semen samples of both sides, while the respective values of the semen parameters were the worst for the samples of the left epididymis (30.70% and 0.90% vs. 42.60% and 2.50% for left and right epididymis, respectively). The velocity variables revealed higher values for the spermatozoa obtained from the right compared to the left epididymis (rapid, medium, slow movement spermatozoa, VCL, VSL, VAP). Regarding CASA kinetic variables related to progressiveness (LIN, STR, BCF), the values were similar for spermatozoa collected from both sides.

The sperm viability values were low for both sides (26.00% and 23.00% for left and right epididymis, respectively). The percentages of spermatozoa with normal morphology were 86.00% and 78.50% for the left and the right epididymis, respectively (Table 2). Among the classified sperm morphological abnormalities, spermatozoa with abnormal tail were predominant (12.50% and 18.00% for the left and the right epididymis, respectively; Table 2). Spermatozoa with fragmented DNA chromatin structure were not detected in both samples.

## 3. Discussion

Ovine varicocele has rarely been diagnosed and has been associated with testicular degeneration and low-quality semen parameters; even published articles about ram varicocele are restricted, and most of them are not recent [7,8,9]. Published data from the 1980s–1990s indicate the testicular mineralization and occluding thrombosis of testicular vessels [8,14]. Moreover, it was described that the bilateral ram varicocele was accompanied by decreased semen quality [9]. In a more recent study conducted in Algeria, the authors investigated the prevalence of testicular pathological conditions in farm animals and found that ram varicocele was the less common lesion observed in that species [7]. Among the 450 rams included in that study, only two rams were diagnosed with varicocele [7]. The results of the present case report are in agreement with these studies, indicating that this pathological condition is associated with testis dysfunction and degradation of ram semen quality [8,15]. The total sperm motility demonstrated lower values of the usually considered limit for the routine application of artificial insemination (>85%). The total sperm motility of the sample collected from the right side was higher than the left one (42.60% vs. 30.00% for right and left side, respectively). This result could possibly be explained by the observed simultaneous left testicular torsion, which probably worsened the laboratory findings compared to the right side. Furthermore, spermatozoa obtained from the right side provided higher abnormal tail values compared to the sperm obtained from the other side. This finding is not easy to explain, since it could be expected that spermatozoa obtained from the side with the greater lesions would have provided the worst values. However, the tail abnormalities are usually attributed to stress effects after semen collection. According to Blom (1983) [16], sperm defects are subdivided into three categories: (1) primary, attributed to the disorder of spermatogenesis; (2) secondary, attributed to disturbances during spermatogenesis and the epididymal transportation of spermatozoa; (3) tertiary, attributed to the improper stressful handling of the semen sample. Based on the description of the tertiary abnormalities, the high percentage of tail defects could be attributed to sperm sensitivity to the laboratory’s technical evaluation handling. In addition, any animal’s disease, and stress as a result of pain, may affect spermatogenesis and epididymal function, as high cortisol levels repress the testosterone concentrations [17], while a temperature shock or stress event during the passage of spermatozoa through the epididymis could lead to high number of tail defects [18]. In any case, for both sides, spermatozoa values with morphological abnormalities were below the accepted limit for the further sperm processing of 25%. Another interesting finding is that no negative influence was observed on sperm morphology and DNA integrity, as we initially expected. Since the swelling in the scrotal area was accidentally noticed by the owner, we could not determine exactly when the lesions were caused. Therefore, we could hypothesize that since sperm morphology and DNA integrity, which are related to spermatogenesis, were not affected, ram varicocele could have been recently caused without affecting the last spermatogenic cycle. The low values of total epididymal sperm motility could be expected, since sperm motility is related to sperm maturation in epididymis. The varicocele, however, could negatively affect epididymis function. Therefore, spermatozoa interaction with epididymal secretions, as well as sperm membrane integrity, could be impaired, and this could be a further explanation for the low values of sperm motility and viability. In the present case, spermatozoa were detected even in the bilateral high-grade varicocele. In contrast, in another case of ram varicocele, which was notably unilateral, azoospermia was noticed [15]. Therefore, we could speculate that, except for the grade, the varicocele is not the only pathological condition related to sub or infertility and, since it is concomitant with other pathological situations, as described by Lacasta et al., 2009 [15], the testicular function could be further impaired. Finally, since rams are often used for natural mating in traditional sheep breeding, varicose could be one of the reasons for the reduction of the fertility rate and the removal of the male.

## 4. Conclusions

In conclusion, the bilateral ram varicocele diagnosed in the present study negatively affected sperm quality, at a level associated with male sub-fertility, but not infertility. In addition, motility and kinematics were mainly affected within the evaluated sperm quality parameters.

## Figures and Tables

**Table 1:** Computer-assisted sperm analyzer (CASA) motility and kinetics evaluation of epididymal ram spermatozoa.

Sperm Kinematics	Epididymal Spermatozoa	
Variable	LE	RE
Total (%)	30.70	42.60
Progr. (%)	0.90	2.50
Rapid (%)	3.30	9.60
Medium (%)	2.60	5.50
Slow (%)	24.80	27.50
VCL (μm/s)	34.40	50.60
VSL (μm/s)	8.10	13.50
VAP (μm/s)	15.70	28.50
ALH (μm)	2.40	2.50
BCF (Hz)	10.60	13.20
LIN (%)	23.50	26.60
STR (%)	51.40	47.30
WOB (%)	45.70	56.30

LE: left epididymis; RE: right epididymis. Total: total motility (%); Progr.: progressive movement spermatozoa (%); rapid/medium/slow: rapid/medium/slow movement spermatozoa (%, 10 < slow < 25 < medium < 45 < rapid μm/s); VCL: curvilinear velocity (μm/s); VSL: straight line velocity (μm/s); VAP: average path velocity (μm/s); ALH: amplitude of lateral head displacement (μm); BCF: beat/cross-frequency (Hz); LIN: linearity (VSL/VCL × 100); STR: straightness (VSL/VAP × 100); WOB: wobble (VAP/VCL × 100).

**Table 2:** Epididymal ram spermatozoa with normal morphology or morphological abnormalities (head (including integrity of acrosome membrane), midpiece, tail, cytoplasmic droplets).

Sperm Morphology	Epididymal Spermatozoa	
Variable	LE	RE
Normal morphology (%)	86.00	78.50
Head (%)	0.75	0.50
Midpiece (%)	0.75	3.00
Tail (%)	12.50	18.00
Cytoplasmic droplets (%)	0.00	0.00

LE: left epididymis; RE: right epididymis. Normal morphology: spermatozoa with normal morphology (%); head/midpiece/tail: spermatozoa with abnormal head/midpiece/tail (%); Cytoplasmic droplets: spermatozoa with cytoplasmic droplet.
